# The prevalence of and risk factors for diabetes mellitus and impaired glucose tolerance among Tibetans in China: a cross-sectional study

**DOI:** 10.18632/oncotarget.21301

**Published:** 2017-09-28

**Authors:** Shaopeng Xu, Qing Wang, Jie Liu, Bo Bian, Xuefang Yu, Xiangdong Yu, Xianjia Ning, Jinghua Wang

**Affiliations:** ^1^ Department of Cardiology, Tianjin Medical University General Hospital, Tianjin 300052, China; ^2^ Department of Neurology, Tianjin Medical University General Hospital, Tianjin 300052, China; ^3^ Department of Epidemiology, Tianjin Neurological Institute & Tianjin Medical University General Hospital, Tianjin 300052, China; ^4^ Tianjin Neurological Institute, Key Laboratory of Post-Neuroinjury Neuro-repair and Regeneration in Central Nervous System, Ministry of Education and Tianjin City, Tianjin 300052, China; ^5^ Central of Clinical Epidemiology, Tianjin Medical University General Hospital, Tianjin 300052, China

**Keywords:** diabetes mellitus, epidemiology, prevalence, risk factors, Tibetans

## Abstract

The prevalence of diabetes mellitus (DM) and impaired glucose tolerance (IGT) has increased worldwide, although their prevalence and determinants among Tibetans are currently unknown. We thus aimed to explore the prevalence of and risk factors for DM and IGT among Tibetans in China. In 2011, 1659 Tibetan adults (aged ≥ 18 years) from Changdu, China, were recruited to this cross-sectional study. They completed a questionnaire and underwent physical examinations and laboratory testing to assess risk factors for DM and IGT. The age-standardized prevalence of DM and IGT among Tibetans was 6.2% and 19.7%, respectively. A higher annual family income, alcohol consumption, and higher fasting plasma glucose (FPG) level were risk factors for DM, with odds ratio (ORs) and 95% confidence intervals (CIs) of 3.48 (1.43–8.48; *P* = 0.006) for those with family incomes of > 1600 USD/year, 3.06 (1.31–7.17; *P* = 0.010) for alcohol consumption, and 13.99 (7.76–25.22; *P* < 0.001) for FPG level. However, altitude was found to be negatively associated with the risk of DM; compared to individuals living at < 3500 meters, the risk of DM decreased by 65% for those living at 3500–3999 meters (*P* = 0.034) and by 89% for those living at ≥ 4000 meters (*P* = 0.015). Age, FPG levels, and low-density lipoprotein cholesterol levels were significantly associated with IGT among Tibetans aged ≥ 18 years. These findings suggest that the prevalence of DM in Tibetans may continue to increase in future decades following rapid economic development, and it is crucial to address the management of conventional risk factors for reducing the disease burden of DM among Tibetans.

## INTRODUCTION

DM has become one of the most important public health issues in the world [1]. The global age-standardized DM prevalence increased from 4.3% in 1980 to 9.0% in 2014 in men and from 5.0% to 7.9% in women [2]. The number of adults with DM worldwide increased from 108 million in 1980 to 422 million in 2014 [2]. Additionally, the burden of DM, in terms of both prevalence and the number of adults with DM, increased at a greater rate in low-income and middle-income countries than in high-income countries [2]. The worldwide cost of DM was 1.31 trillion USD, or 1.8% of the global gross domestic product (GDP), in 2015 [3].

A recent study estimated that the prevalence of DM was 11.1–12.3% in middle-income countries and that the prevalence varied across different countries [4]. Half of adults with DM in 2014 lived in five countries: China, India, the USA, Brazil, and Indonesia [2]. Moreover, the prevalence of DM has increased rapidly in China, with a considerable subsequent burden [5]. The prevalence of DM was 2.6% in 2002, and it reached 9.7% in 2012 according to the Report on the Status of Nutrition and Chronic Diseases of Chinese residents [6]. A recent study among Tibetans in China reported that the prevalence of DM was 2.9% [2].

However, these studies were conducted in a population residing at the same altitude. Additionally, these studies reached conclusions based on a small population living at base levels and excluded lamas, and some laboratory testing items were not included in these studies. Thus, the prevalence of and risk factors for DM among Tibetans residing at different altitudes, including lamas, are currently unknown.

We thus performed a population-based survey among Tibetans living in China that included lamas and Tibetans living at different altitudes. We aimed to assess the prevalence of and risk factors for DM in Tibet, China.

## RESULTS

### Descriptive characteristics of participants

A total of 1960 residents completed the study survey and examinations, for a response rate of 90.1%. Of those, 1659 Tibetans were included in the present study, after excluding 134 residents without complete demographic data and 167 residents of other ethnicities. Of these, there were 822 (49.5%) men and 837 (50.5%) women. The mean age was 44 years overall, 41.45 years in men and 46.51 years in women; 53% of participants were under 45 years old. The education levels were low both in men and in women, with a 60% illiteracy rate overall. Among all participants, 56.9% had an family income of < 800 USD/year; 59.5% of participants were farmers or herdsmen; and 56% of individuals lived in rural or pastoral areas (Table 1).

**Table 1:** The demographic characteristics of participants in Tibetans

Characteristics	Men (*n* = 822)	Women (*n*= 837)	Total (*n* = 1659)
Age, years, means (SD)	41.45(15.01)	46.51(15.05)	44.00(15.24)
Age group, years, n (%):			
18–34	299 (36.38)	194 (23.18)	493 (29.72)
35–44	194 (23.60)	193 (23.06)	387 (23.33)
45–54	161 (19.59)	201 (24.01)	362 (21.82)
55–64	105 (12.77)	138 (16.49)	243 (14.65)
≥ 65	63 (7.66)	111 (13.26)	174 (10.48)
Education, years, n (%):			
0	394 (47.93)	607 (72.52)	1001 (60.34)
1–6	330 (40.15)	102 (12.19)	432 (26.04)
7–9	46 (5.60)	43 (5.14)	89 (5.36)
10–12	26 (3.16)	38 (4.54)	64 (3.86)
> 12	26 (3.16)	47 (5.61)	73 (4.40)
Family income yearly, USD:			
< 800	508( 61.80)	436 (52.09)	944 (56.90)
800–1599	145 (17.64)	162 (19.36)	307 (18.51)
> 1600	169 (20.56)	239 (28.55)	408 (24.59)
Occupation, n (%):			
Officer	56 (6.81)	93 (11.11)	149 (8.98)
Workers	26 (3.16)	15 (1.79)	41 (2.47)
Farmers	334 (40.63)	582 (69.53)	916 (55.21)
Herdsman	31 (3.77)	40 (4.78)	71 (4.28)
Shaman	337 (41.00)	7 (0.84)	344 (20.74)
Others	38 (4.63)	100 (11.95)	138 (8.32)
Altitude, meter, n (%):			
< 3500	444 (54.0)	463 (55.3)	907 (54.7)
3500–3999	266 (32.4)	334 (39.9)	600 (36.2)
≥ 4000	112 (13.6)	40 (4.8)	152 (9.1)
Residence, n (%):			
Urban	144 (17.52)	251 (29.98)	395 (23.81)
Rural	322 (39.17)	539 (64.40)	861 (51.90)
Pastoral area	31 (3.77)	44 (5.26)	75 (4.52)
Temple	325 (39.5)	3 (0.36)	328 (19.77)

### Associations of DM and IGT with conventional risk factors in the univariate analysis

The age-standardized prevalences of DM and IGT were 6.2% and 19.7% overall, 7.9% and 17.4% in men, 4.9% and 18.8% in women, respectively; there was a significantly higher prevalence of DM in men than in women (*P* = 0.012). The DM prevalence increased significantly with increasing age, education level, and annual family income; however, the opposite trends were observed for education level and altitude. Moreover, the age-standardized prevalence of DM was more likely to be high in those with hypertension, obesity, dyslipidemia, and/or alcohol consumers. The prevalence of IGT significantly increased with age (Table 2). Moreover, the age-standardized prevalences of DM and IGT were associated with the levels of BMI, TC, TG, HDL-C, and FPG (Table 3).

**Table 2:** The association of DM and IGT with related factors among Tibetans aged ≥ 18 years in univariate analysis

Demographical features	DM		IGT	
	Rate (95%CI)	*P*	Rate (95%CI)	*P*
Gender:		0.012		0.199
Men	7.9 (6.0–9.8)		17.4 (14.7–20.0)	
Women	4.9 (3.4–6.3)		19.7 (17.7–21.7)	
Total	6.2 (5.0–7.4)		18.8 (16.0–21.4)	
Age group, years:		< 0.001		< 0.001
18–34	1.9 (0–3.9)		9.8 (7.1–12.5)	
35–44	4.8 (2.6–6.9)		21.5 (17.3–25.6)	
45– 54	8.0 (5.2–10.8)		32.0 (27.1–36.9)	
55–64	13.3 (9.0–17.7)		24.5 (18.9–30.0)	
≥ 65	13.3 (6.9–19.6)		27.7 (20.9–34.5)	
Education level, years:		< 0.001		0.704
0	5.2 (3.8–6.7)		15.8 (15.5–20.1)	
1–6	7.3 (4.7–9.8)		17.5 (13.8–21.1)	
≥ 7	11.9 (7.6–16.2)		16.1 (11.2–20.9)	
Family income yearly:		< 0.001		0.450
< 800	3.9 (2.7–5.2)		17.7 (15.2–20.2)	
800–1599	5.3 (2.8–7.9)		16.1 (12.0–20.3)	
≥1600	13.1 (9.8–16.4)		19.9 (16.0–23.8)	
Altitude, meter:		0.050		0.333
< 3500	7.0 (5.3–8.7)		16.7 (14.1–18.8)	
3500–3999	5.3 (3.5–7.1)		21.0 (17.7–24.3)	
≥ 4000	3.2 (0.4–6.0)		15.8 (10.0–21.6)	
Residence:		0.001		0.379
Urban	9.5 (6.6–12.4)		14.8 (11.2–18.3)	
Rural/Pastoral area	5.2 (3.8–6.7)		19.9 (17.3–22.5)	
Temple	3.8 (1.7–6.0)		16.7 (12.6–20.8)	
Hypertension:		0.002		0.244
Yes	7.2 (5.6–8.7)		19.3 (17.2–22.0)	
No	3.1 (1.7–4.5)		16.6 (13.5–19.7)	
Obesity:		0.001		0.130
Yes	10.7 (7.0–14.4)		22.5 (17.6–27.5)	
No	5.0 (4.9–7.3)		17.8 (13.3–22.3)	
Dyslipidemia:		< 0.001		0.186
Yes	9.1 (6.9–11.3)		19.6 (16.7–22.6)	
No	3.3 (2.2–4.5)		16.7 (14.3–19.2)	
Smoking:		0.895		0.496
Yes	7.2 (2.0–12.5)		15.0 (7.8–22.3)	
No	6.0 (4.8–7.2)		18.2 (16.2–20.2)	
Alcohol consumption:		0.004		0.182
Yes	12.2 (7.0–17.3)		22.7 (16.1–29.3)	
No	5.5 (4.3–6.6)		17.6 (15.6–19.6)	

**Table 3:** The association of DM and IGT with the measurements among Tibetans aged ≥ 18 years in univariate analysis

Disease History	DM		IGT	
	means (SD)	*P*	means (SD)	*P*
BMI:		< 0.001		0.001
Yes	26.53 (4.43)		24.56 (4.03)	
No	23.75 (3.85)		23.75 (3.85)	
TC:		< 0.001		0.018
Yes	5.54 (1.27)		4.94 (1.16)	
No	4.53 (1.03)		4.53 (1.03)	
TG:		< 0.001		0.011
Yes	2.17 (2.94)		1.14 (0.92)	
No	1.01 (0.78)		1.01 (0.78)	
HDL-C:		0.550		0.410
Yes	1.50 (0.48)		1.55 (0.44)	
No	1.52 (0.44)		1.52 (0.44)	
LDL-C:		< 0.001		< 0.001
Yes	2.79 (0.97)		2.61 (0.91)	
No	2.29 (0.76)		2.29 (0.76)	
FPG:		< 0.001		< 0.001
Yes	8.01 (4.34)		5.44 (0.84)	
No	4.86 (0.67)		4.86 (0.67)	

### Risk factors associated with DM and IGT among Tibetans aged ≥ 18 years in the multivariate analysis

Table 4 shows that annual family income, altitude, alcohol consumption, and FPG levels were significantly associated with DM among Tibetans aged ≥ 18 years. A higher annual family income, alcohol consumption, and higher FPG levels were the risk factors for DM, with ORs (95% CIs) of 3.48 (1.43–8.48; *P* = 0.006) for those with a family income of >1600 USD/year, 3.06 (1.31–7.17; *P* = 0.010) for those who consumed alcohol, and 13.99 (7.76–25.22; *P* < 0.001) for higher FPG levels. However, altitude was found to be negatively associated with the risk of DM; compared to individuals living at < 3500 meters, the risk of DM decreased by 65% for those living at 3500–3999 meters (*P* = 0.034) and by 89% for living at ≥ 4000 meters (*P* = 0.015).

**Table 4:** Associated risk factors of DM among Tibetans aged ≥ 18 years in the multivariate analysis

Risk factors	Reference	OR	95%CI
Men	Women	1.32	0.66~2.64
Age:	18–34 years		
35–44 years		0.79	0.27~2.31
45– 54 years		0.62	0.21~1.85
55–64 years		1.06	1.35~3.24
≥ 65 years		2.17	0.68~6.90
Education level	0 Years		
1–6 Years		0.68	0.28~1.65
≥ 7 Years		0.53	0.18~1.57
Family income yearly	< 800 USD		
800–1599 USD		1.65	0.62~4.35
> 1600 USD		**3.48**	**1.43~8.48**
Altitude:	< 3500 meters		
3500–3999 meter		**0.35**	**0.14~0.93**
≥ 4000 meter		**0.11**	**0.02~0.66**
Residence	Urban		
Rural/ Pastoral area		1.17	0.42~3.27
Temple		0.95	0.21~4.32
Hypertension	No	0.58	0.25~1.34
Obesity	No	0.83	0.27~2.59
Dyslipidemia	No	1.02	0.44~2.39
Alcohol consumption	No	**3.06**	**1.31~7.17**
BMI	—	1.12	0.98~1.28
TC	—	0.77	0.41~1.45
TG	—	1.23	0.89~1.70
LDL-C	—	1.99	0.92~4.30
FPG	—	**13.99**	**7.76~25.22**

Age and the levels of FPG and LDL-C were independent risk factors for IGT among Tibetans aged ≥18 years. Compared to individuals aged 18–34 years, the risk of IGT increased by 95% for those in the 35–44 years group, by 86% for those aged 45–54 years, by 1.02-fold for those aged 55–64 years, and by 1.54-fold for those aged 65 years and older (all *P* < 0.05). The corresponding ORs (95% CIs) were 1.41 (1.04–1.91; *P* = 0.025) for LDL-C, and 3.38 (2.69–4.23; *P* < 0.001) for FPG, respectively (Table 5).

**Table 5:** Associated risk factors of IGT among Tibetans aged ≥ 18 years in the multivariate analysis

Risk factors	Reference	OR	95%CI
Age:	18–34 years		
35–44 years		**1.95**	**1.28~2.97**
45– 54 years		**1.86**	**1.20~2.87**
55–64 years		**2.02**	**1.26~3.24**
≥ 65 years		**2.54**	**1.53~4.21**
BMI	—	1.01	0.97~1.05
TC	—	0.98	0.76~1.26
TG	—	1.09	0.91~1.30
LDL-C	—	**1.41**	**1.04~1.91**
FPG	—	**3.38**	**2.69~4.23**

## DISCUSSION

This is the first study to report the prevalence of and risk factors for DM and IGT among Tibetans in China. The age-standardized prevalences of DM and IGT were 6.2% and 19.7% overall, respectively. Family income, altitude, alcohol consumption, and FPG levels were factors that were associated with DM among Tibetans aged ≥ 18 years. There was an increased risk of DM in residents with a higher family income (> 1600 USD/year compared to <800 USD/year), those who were alcohol drinkers, and those who had elevated levels of FPG. However, the risk of DM decreased with an increase in altitude; compared to residents living at < 3500 miles, the risk of DM decreased by 65% and 89% in those living at 3500–3999 meters and ≥ 4000 meters, respectively. Moreover, older age and high levels of LDL-C and FPG were independent risk factors for IGT.

In 2014, the prevalence of DM was reported to be 9.0% for men and 7.9% for women worldwide [2]. The reported prevalence of DM in China has an extensive range. A national survey revealed that the prevalences of DM and IGT among adults aged 20 years and older in 2010 were 9.7% (10.6% for men and 8.8% for women) and 15.5% (16.1% for men and 14.9% for women), respectively [7]. The findings from a nationally representative cross-sectional survey on the ethnic variation of DM and IGT prevalence in China indicated that the overall standardized prevalence of diabetes in Chinese adults was estimated at 10.9% (95% CI, 10.4–11.5%) in 2013, or 10.2% (95% CI, 9.7–10.7%) for women and 11.7% (95% CI, 10.9–12.4%) for men; the estimated prevalence of IGT was 35.7% (95% CI, 34.1–37.4%) for the overall population, or 35.0% (95% CI, 33.4–36.7%) for women and 36.4% (95% CI, 34.6–38.2%) for men; Tibetan and Muslim Chinese individuals had a significantly lower prevalence of diabetes than did Han participants [8]. The overall age-standardized prevalence of type 2 diabetes, combining urban and rural areas, was 12.6% in 2009 among Chinese adults aged 35–74 years in Shanghai [9], which was considerably higher than that in a previous report [7]. However, a relatively lower prevalence of DM was found in other regions, such as in Hunan (1.5%), Guizhou (1.9%), Guangxi (2.5%), and Hubei (2.7%) [10]. Consistent with these results, the age-standardized prevalences of DM and IGT were 6.2% and 18.8% overall, 7.9% and 17.4% for men, 4.9% and 19.7% for women among Tibetans aged ≥ 18 years in China, respectively. The specific lifestyle in this population may explain the lower prevalence of DM.

Age has previously been found to be associated with a higher prevalence of DM [11]. However, in this study, age was associated with the risk of IGT but not with DM. An increasingly sedentary lifestyle with increasing age could contribute to the age-related increases in the prevalence of IGT. The exact mechanisms of the age difference between DM and IGT prevalence would need to explore deeply.

It has been reported that, compared to residents at sea level, residents living at high altitudes have lower fasting glucose levels [12–16] and better glucose tolerance [17]. A recent study from the United States revealed that the risk of DM among adults living at high altitudes (1500–3500 meters) decreased by 12% compared to that for those living at < 500 meters, after adjusting for age, sex, BMI, ethnicity, and lifestyle-related factors [18]. In this study, the prevalence of DM decreased as altitude increased. The exact mechanisms underlying the lower prevalence of DM at high altitudes are unclear. In order to improve adaptation to hypoxic conditions, glucose oxidation and glycolysis replace fatty acid oxidation; this may contribute to the lower risk of DM in residents living at higher altitudes [19]. A previous study reported a high-frequency missense mutation in the *EGLN1* gene, which encodes prolyl hydroxylase 2 (PHD2), among Tibetans, which prevents the development of polycythemia in these high-altitude dwellers [20] and that was unrelated to any increased hemoglobin oxygen affinity or any hemoglobin variants for these adaptive changes [21]. Another PPARA haplotype that encodes the nuclear peroxisome proliferators activated receptor α (which regulates fatty acid metabolism and in turn hypoxia-inducible factors) is correlated with a possible decrease in the activity of fatty acid oxidation [22]. These data suggest that high-altitude adaptations may offer protection from DM at high altitudes but that the risk of DM would increase at lower altitudes [23].

The association between alcohol drinking and the risk of DM has been established. Previous studies showed that alcohol consumption exhibits a U-shaped relationship with the risk of T2DM in both men and women, with two alcoholic drinks per day (~50 g/day) increasing the relative risk of DM [24, 25]. Moderate alcohol intake (1–3 drinks/day) was inversely related to T2DM risk in adults [26]. Alcohol consumption was found to protect against DM at a level of 24 g/day for women and 22 g/day for men but it became a risk factor at levels of > 50 g/day for women and >60 g/day for men [25]. Severe alcohol consumption was associated with elevated FPG in adults aged 16 to 43 years [27]. Similar to those studies, alcohol consumption was found to be an independent risk factor for DM in this study, after adjustment for other conventional risk factors; the risk of DM in alcohol drinkers increased by more than two-fold compared to that for non-drinkers. However, the dose-reaction relation between alcohol consumption and the risk of DM was not assessed in this study.

The association of the LDL-C level and the presence of IGT has been undetermined. Several studies have demonstrated that the prevalence of IGT was not associated with the level of LDL-C [28, 29], but hypertriglyceridemia emerged as a significant risk factor for DM and IGT in an Asian population [30]. In contrast to these previous studies, the level of LDL-C in plasma, not the TG level, was significantly associated with the risk of IGT among Tibetans aged ≥ 18 years in China in this study. These findings imply that there was potential combined effect between risk factors.

There were several limitations to this study. First, it was a cross-sectional study; thus, it cannot determine causal conclusions. Second, the population was selected in one area; however, the four-stage randomly stratified cluster sampling method was used to make the study population representative. Third, all participants were from higher altitudes (> 3200 meters), and we did not include a population at a lower altitude for purposes of comparison. However, the subgroup analyses were stratified according to altitude (< 3500 meters, 3500–3999 meters, and ≥ 4000 meters). Fourth, the prevalence of DM was associated with annual family income, but as Tibet is an undeveloped region, the level of economic development in this population was low overall. Thus, although the level of income in Tibetans is relatively low, annual family income was stratified into three groups (< 800 USD/year, 800–1600 USD/year, and ≥ 1600 USD/year). Moreover, the dose-reaction relation between alcohol consumption and the risk of DM was not assessed in this study, which may have affected the evaluation of the association of alcohol drinking with DM. Finally, this study was conducted in 2011; thus, there is a 6-year interval, so the information it provides on the prevalence of DM and IGT is not up-to-date. Thus, it is necessary to perform a more current study of the trends for risk factors among this population.

This is the first study to report the prevalence of and risk factors for DM and IGT among Tibetans in China. A higher prevalence of IGT was observed among residents living in rural or pastoral areas, although there was a lower prevalence of IGT overall. China is a developing country undergoing a transition from an agricultural society to an industrial society. The Tibet Autonomous Region is the least urbanized area in China, and the level of economic development in this population is lower overall. According to our study, the prevalence of DM in Tibetans may increase as the economy develops. A low altitude and elevated FPG levels were common risk factors for DM and IGT among Tibetans aged ≥18 years. However, a higher family income and alcohol drinking increased the risk of DM; older age and elevated levels of LDL-C were independent risk factors for IGT. These findings suggest that the prevalence of DM in Tibetans may continue to increase in future decades following rapid economic development, and it is crucial to address the management of conventional risk factors in order to reduce the disease burden of DM among Tibetans.

## MATERIALS AND METHODS

### Study population

The study design has been described previously [31]. In brief, the study population was recruited from the Changdu region of the Tibet Autonomous Region of China from September 2010 to June 2011. There are 11 counties, including 142 townships and 11 central temples, in the Changdu region, with altitudes ranging from 3200 to 4500 meters. More than 95% of residents are Tibetan.

A four-stage randomly stratified cluster sampling method was used to select a representative sample of the Tibetan population in China. First, all 11 counties in the Changdu region were stratified into three groups according to altitude: < 3500 meters, 3500–4000 meters, and > 4000 meters. Second, one county was selected from each altitude group, which included three counties. Third, four townships from each selected county were selected, for a total of 12 townships. Fourth, three villages or neighborhoods from each selected township were selected, resulting in a total of 36 villages or neighborhoods. Moreover, one central temple was selected in each county; all qualified lamas were recruited in the present study. Finally, all residents aged ≥ 18 years from the selected 31 villages, 5 neighborhoods, and 3 central temples were recruited in this survey.

The ethics committee of Changdu Region People's Hospital, Tibet approved the study, and written informed consent was obtained from all participants during recruitment.

### Study content

In 2011, 1659 Tibetan adults aged ≥18 years from Changdu, China were recruited to this cross-sectional study. The questionnaire, physical examinations, and laboratory testing were completed, and the prevalence of DM and risk factors, including hypertension, obesity, dyslipidemia, and current smoking, were evaluated. The association between the DM risk factors and demographic characteristics, as well as geographic altitude, was assessed.

Detailed information evaluated included demographic information, such as sex, age group (18–34 years, 35–44 years, 45–54 years, 55–64 years, ≥ 65 years), education level (0 years, 1–6 years, ≥ 7 years), and occupation (officer, workers, farmers, herdsman, lama, others); socioeconomic status, including yearly family income (< 800 USD/year, 800–1599 USD/year, 1600 USD/year); geographic characteristics, including altitude (< 3500 meters, 3500–3999 meters, and ≥ 4000 meters), and residence (township, rural, temple); and lifestyle factors, including cigarette smoking (no, yes) and alcohol consumption (no, yes). The physical examinations included measurement of blood pressure (BP); body height and weight; and circumferences of the waist, hips, and abdomen. Additionally, all participants took the fasting plasma glucose (FPG) and oral glucose tolerance test (OGTT), and blood samples were obtained to determine fasting glucose, total cholesterol (TC), triglycerides (TG), high-density lipoprotein cholesterol (HDL-C), and low-density lipoprotein cholesterol (LDL-C) levels.

### Definitions of DM and impaired glucose tolerance (IGT)

DM was defined as a FPG level ≥ 7.0 mmol/L, OGTT results of ≥ 11.11 mmol/L, a self-reported history of DM, or current treatment with antidiabetic medication [32]. IGT was defined as 2-hour glucose levels of 7.8–11.1 mmol/L (140–200 mg/dL) on the 75-g OGTT [33].

### Dyslipidemia and hypertension

Hypertension was defined as an average systolic blood pressure (BP) of ≥ 140 mmHg, an average diastolic BP of ≥ 90 mmHg, and/or self-reported current treatment for hypertension with antihypertensive medication [34].

Dyslipidemia was defined as self-reported current treatment with cholesterol-lowering medication or having one or more of the following levels: TC ≥ 5.2 mmol/L, TG ≥ 1.7 mmol/L, HDL-C < 1.04 mmol/L, or LDL-C ≥ 3.4 mmol/L [35].

### Body mass index (BMI) and obesity definitions

BMI was calculated as weight (in kilograms) divided by the square of height (in meters). According to standard criteria in Chinese adults, overweight and obesity were defined as BMIs of 24.0–27.9 kg/m^2^ and 28.0 kg/m^2^, respectively [36].

### Data collection

Surveys were conducted in community health stations through face-to-face interviews, and physical examinations were performed by trained research staff guided by epidemiology professionals. A few participants completed the survey at home. A standardized questionnaire was administered in this survey.

### Statistical analysis

Data are presented as means (standard deviations) for continuous variables (including age, BMI, TC, TG, HDL-C, LDL-C, and FPG), and as number (percentages) for categorical variables (including sex, age group, education level, annual family income, altitude group, residence group, and previous disease histories). The age-standardized prevalences of DM and IGT were calculated with the direct method using the world standard population [37] and are presented as rates with 95% confidential intervals (CIs). Differences in continuous variables between two groups were compared using Student's *t*-test. Differences in categorical variables were compared using Pearson's chi-squared test for two groups and the linear-by-linear association for trends. Risk factors for DM and IGT were assessed by the chi-squared test and Student's *t*-test in the univariate analysis. A logistic regression analysis was performed to assess the risk factors for DM and IGT in the multivariate analysis, with the dependent variable set as DM or IGT (as dichotomous variables) and the independent variables set as factors determined to be statistically significant in the univariate analysis, respectively. The results of the multivariate analysis are presented as adjusted odds ratios (ORs) with 95% confidence intervals (CIs) for the associated factors in the univariate analysis, respectively. Statistical significance was defined as a two-tailed *P* < 0.05. SPSS version 15.0 for Windows (SPSS Inc., Chicago, IL, USA) was used for the analyses.

## Abbreviations

DM: diabetes mellitus
IGT: impaired glucose tolerance
FPG: fasting plasma glucose
OR: odds ratio
CI: confidence interval
PHD2: prolyl hydroxylase 2
BMI: body mass index
BP: blood pressure
OGTT: oral glucose tolerance test
TC: total cholesterol
TG: triglycerides
HDL-C: high-density lipoprotein cholesterol
LDL-C: low-density lipoprotein cholesterol
